# Phage-antibiotic synergy attenuates *Acinetobacter baumannii* resistance in refractory pneumonia: a precision therapeutic case

**DOI:** 10.3389/fcimb.2026.1851410

**Published:** 2026-07-09

**Authors:** Lei Zhang, Kexin Yan, Pengli Xu, Yanyu Xiao, Cheng Guo, Guiqin Dai, Jie Lin, Deliang Liu, Man Rao, Zhiqiang Lin, Pengfei Zhao, Mingbin Zheng, Yang Zhou, Hongzhou Lu

**Affiliations:** 1 National Clinical Research Center for Infectious Disease, Shenzhen Third People’s Hospital, Southern University of Science and Technology, Shenzhen, China; 2 School of Medicine, Shenzhen University, Shenzhen, China; 3 School of Public Health, Sun Yat-Sen University, Guangzhou, China; 4 Guangdong Key Lab for Diagnosis & Treatment of Emerging Infectious Diseases, Shenzhen Third People’s Hospital, Shenzhen, China; 5 The Affiliated Dongguan Songshan Lake Central Hospital, Guangdong Medical University, Dongguan, China; 6 Molecular Biology Research Center & Center for Medical Genetics, School of Life Sciences, Central South University, Changsha, China

**Keywords:** *Acinetobacter baumannii*, antibiotics, antimicrobial resistance genes, bacteriophage, extensive drug resistance

## Abstract

Extensively drug-resistant (XDR) *Acinetobacter baumannii* pneumonia carries severe pneumonia, respiratory failure and high mortality, showing limited therapeutic options in critically ill patients. Although bacteriophage (phage) therapy represents a promising alternative against drug-resistant infections, its clinical use remains largely empirical. Here, we reported a systematically planned phage-antibiotic combination strategy in a critically ill patient with refractory XDR *A. baumannii* pneumonia. A virulent phage targeting the patient-derived strain was isolated from hospital wastewater and classified within the class *Caudoviricetes*, with no virulence, toxin, or antibiotic resistance genes. *In vitro* time-kill assays showed that phage monotherapy failed to persistently suppress bacteria proliferation, whereas phage-antibiotic therapy achieved synergistic inhibition of *A. baumannii* growth for over 48 h. The patient received nebulized phage therapy (5 × 10^9^ PFU/mL twice daily) combined with intravenous fosfomycin (8 g, every 8 hours), amikacin (0.2 g, every 12 hours), and polymyxin B (500, 000 U, every 12 hours). Clinically, treatment was associated with rapid normalization of arterial carbon dioxide tension (PaCO2), clearance of A. baumannii sputum cultures by day 4, declining inflammatory markers, and no treatment-related toxicity.Longitudinal metagenomic sequencing further revealed approximately 52-fold reduction in pathogen abundance and significant decrease of *A. baumannii*-associated antimicrobial resistance genes (ARGs) in the lung, highlighting the potential of precision phage-antibiotic therapy for recalcitrant XDR bacterial infections.

## 1 Introduction

Extensively drug-resistant (XDR) *Acinetobacter baumannii* (*A. baumannii*) infection is a major cause of hospital-acquired pneumonia in critically ill patients, which results in severe pneumonia, respiratory failure and high mortality (Fitzpatrick et al., 2015; Tiku et al., 2022). Although antibiotics remain the standard strategy of therapy, achieving durable bacterial eradication with these conventional agents is increasingly difficult (Tacconelli et al., 2018). This challenge arises from the limited agents with reliable *in vivo* activity against XDR strains, and dose-dependent toxicity further narrows the therapeutic window (Wright et al., 2016; Czuban et al., 2018). Consequently, antibiotics treatment outcomes to XDR *A. baumannii* infection are often poor, and effective disease management remains a major clinical challenge (Liepa et al., 2022). These persistent limitations underscore the urgent need for alternative or adjunctive strategies to overcome the constraints of conventional antibiotics (Khosravi et al., 2014).

Phage therapy has emerged as a promising strategy for treating drug-resistant bacterial infections (Seniya and Jain, 2022). Virulent phage can specifically infect and replicate within host bacteria, enabling targeted killing of multidrug-resistant pathogens (Dedrick et al., 2019). However, phage monotherapy is constrained by narrow host specificity and the rapid evolution of phage-resistant variants (Li et al., 2020). Through complementary killing mechanisms and broader antimicrobial coverage, phage-antibiotic combinational therapy has therefore been proposed to enhance antibacterial efficacy, restrict evolutionary escape and delay resistance emergence (Gordillo Altamirano et al., 2022; Rosenkilde et al., 2019). However, there is a lack of strong clinical evidence for using phage-antibiotic combinations against XDR infections in critically ill patients (Fanaei Pirlar et al., 2022), and it is essential to clarify whether these interactions are synergistic or antagonistic *in vivo*.

Here, we reported synergistic phage-antibiotic therapy for a critically ill patient with refractory XDR *A.baumannii* pneumonia. A virulent phage against the patient’s clinical bacteria isolate was screened from environmental sources, and its functionality and genomic safety were systematically characterized. An optimized phage-antibiotic combination regimen was identified using *in vitro* time-kill assays and subsequently applied clinically for the treatment of XDR *A. baumannii* infection. Clinically, the combined therapy was associated with rapid improvement in respiratory function, sustained bacterial clearance, reduced systemic inflammation, and a favorable safety profile without evident organ toxicity. Longitudinal metagenomic sequencing of respiratory specimens was performed to monitor pathogen abundance, phage dynamics, and antimicrobial resistance gene profiles throughout treatment. Our findings supported precision phage-antibiotic combinations as a rationally designed strategy for managing life-threatening XDR bacterial infections, with potential benefits in reducing the associated genomic resistance burden.

## 2 Methods

### 2.1 Bacterial strain

The clinical *A. baumannii* isolate, obtained from the patient’s sputum culture, was maintained in Luria-Bertani (LB) broth (Solarbio, Beijing, CHN) and stored in 15% glycerol at -80 °C. Antimicrobial susceptibility testing was performed using the broth microdilution method according to the Clinical and Laboratory Standards Institute (CLSI) guidelines. Minimum inhibitory concentrations (MICs) were determined after incubation at 37 °C for 20 h.

### 2.2 Phage isolation and preparation

Phages were obtained from hospital wastewater using a clinically derived XDR *A. baumannii* isolate as the propagation host. Briefly, wastewater samples were filtered through 0.22 μm membrane filters (Merck Millipore, MA, USA). For phage enrichment, 100 μL of the exponential-phase culture of the host *A. baumannii* strain was inoculated into a mixture of 10 mL of the filtered wastewater sample and 5 mL of 2 × LB broth, followed by overnight incubation at 37 °C with continuous agitation at 220 rpm. The culture was then centrifuged and filtered through 0.22 μm membrane to remove bacterial debris. The enriched lysate was subsequently subjected to purification using the double-layer agar technique as previously reported (Chen et al., 2018). A single clear plaque was picked into SM buffer (Thermo Scientific, MA, USA) and re-isolated using the double-layer agar method. This purification was repeated for five consecutive times to ensure purity. Host range was evaluated by spot assay against a panel of clinical *A. baumannii* isolates based on plaque formation or clear lysis zones.

The suspension was further treated with Triton X-100 (10% stock solution; final concentration 5%) and ultrafiltrated against 0.9% NaCl using Ultramem 50 membranes (Membrane Solutions Co., Ltd., Shanghai, CHN) to eliminate residual impurities. Lastly, the purified phage suspension was sterilized using 0.22 μm filters, and phage titers were quantified by plaque assay, while endotoxin levels were determined using an End-point Chromogenic Endotoxin Test Kit (Bioendo, Xiamen, CHN). The final preparation was stored at 4 °C before clinical use.

### 2.3 Phage characterization and genomic analysis

Phage morphology was examined by transmission electron microscopy (TEM) following negative staining. Briefly, 5 μL of phage suspension was absorbed onto a 150-mesh carbon-coated copper grid for 2 to 5 min by pipetting the suspension onto the grid. After removing excess liquid, the grid was negatively stained with 1% phosphotungstic acid for 1 to 2 min and visualized using a transmission electron microscope (HT7800, Hitachi, Tokyo, JPN).

Phage genomic DNA was extracted using a phage genomic DNA extraction kit (Aidlab, Beijing, CHN) according to the manufacturer’s instructions (Chen et al., 2020). Whole-genome sequencing was performed by Guangzhou Forevergen Biotechnology Co., Ltd. (Guangzhou, CHN) using the Illumina HiSeq platform (Illumina Inc., CA, USA). Raw reads were quality-filtered and assembled *de novo* using SPAdes v4.0 (https://github.com/ablab/spades), followed by polishing with Pilon (Pilon, RRID: SCR_014731). The assembled contig was further evaluated using ViralVerify (Antipov et al., 2020) to confirm its viral origin and assessed as complete using viralComplete (https://github.com/ablab/viralComplete). Open reading frames were predicted and annotated using RAST (https://rast.nmpdr.org), with additional functional annotation performed by BLASTp (BLASTP, RRID: SCR_001010) searches against public protein databases, including the NCBI non-redundant protein database, Pfam, and UniProt. Genomic safety was evaluated by screening ORFs against CARD, ResFinder, MEGARes, and VFDB (E-value ≤ 1 × 10^-5^, coverage ≥ 70%, identity ≥ 30%) to exclude virulence factors, toxins, and resistance genes. Lysogeny-associated genes were screened using PHASTEST (https://phastest.ca/) against the Pfam database. Phage taxonomic classification was performedxthrough sequence similarity analysis using BLASTn (https://blast.ncbi.nlm.nih.gov) and whole-genome average nucleotide identity (wgANI) calculated with OrthoANIu (https://www.ezbiocloud.net/tools/orthoaniu), according to the species demarcation threshold of 95% wgANI recommended by the International Committee on Taxonomy of Viruses (ICTV) (Aldeguer-Riquelme et al., 2024). A circular map of the phage was visualized using the CGView Server (Grant and Stothard, 2008).

### 2.4 *In vitro* antibacterial activity assays

To evaluate the synergistic effects of phage and antibiotics on bacterial growth, bacterial cells (10^5^ CFU/well), antibiotics, and phage multiplicity of infection (MOI) = 0.1 were co-incubated in 96-well plates (Corning, NY, USA) with 200 μL of LB medium per well. Antibiotics were used at 0.25 × MIC, including fosfomycin (128 mg/L), amikacin (128 mg/L), and polymyxin B (0.5 mg/L). Blank medium controls consisting 200 μL of LB were included. Control wells contained only 10^5^ CFU/well of bacteria. Bacterial growth was monitored every 15 min for 48 h at 600 nm using a Synergy H2 microplate reader (BioTek, VT, USA). To quantify synergy, we adopted the highest single agent (HSA) model based on the area under the growth curve (AUC). The synergy score S_HSA_ was defined as:


$$S_{HSA}=\min(\frac{AUC_{P}}{AUC_{0}},\frac{AUC_{A}}{AUC_{0}})-\frac{AUC_{AP}}{AUC_{0}}$$

where AUC_0_, AUC_P_, AUC_A_, and AUC_AP_ are the areas under the curve for the untreated control, phage alone, antibiotic alone, and the combination, respectively. A positive (>0) SHSA indicated synergy, a value near zero indicated additivity, and a negative (<0) value indicates antagonism (Tchatchiashvili et al., 2026).

### 2.5 Clinical treatment and monitoring

A critically ill patient with refractory XDR *A. baumannii* pneumonia, who showed a poor response to prolonged treatment with multiple antibiotics, was enrolled for compassionate use treatment. The treatment regimen consisted of adjunctive nebulized phage administration combined with intravenous antibiotics, including fosfomycin (8 g, every 8 hours), amikacin (0.2 g, every 12 hours), and polymyxin B (500, 000 U, every 12 hours). Purified phage was administered *via* inhalation twice daily at 5 × 10^9^ PFU/mL. Clinical laboratory parameters were collected, including arterial carbon dioxide tension (PaCO_2_) measured using an i-STAT system (Abbott, Shanghai, CHN), white blood cell (WBC) count, neutrophil count (NEU), lymphocyte count (LYM), platelet count (PLT), hemoglobin (HB), C-reactive protein (CRP), and erythrocyte sedimentation rate (ESR) were measured using a BC-6800 Plus hematology analyzer (Mindray, Shenzhen, CHN), and alanine aminotransferase (ALT), aspartate aminotransferase (AST), and serum creatinine were measured using a BS-2000M biochemical analyzer (Mindray, Shenzhen, CHN). Microbiological clearance was assessed by serial sputum cultures.

### 2.6 Metagenomic sequencing of sputum samples

Sputum samples were longitudinally collected on days -1, 0, 3, and 9. Samples were preserved in DNA/RNA Shield (Zymo Research, CA, USA), and microbial DNA was extracted using a commercial kit (Zymo Research, CA, USA) for subsequent metagenomic sequencing. Raw FASTQ files were processed using the Chan Zuckerberg ID (CZ ID, formerly IDseq) mNGS pipeline (version 8.3), including quality control, host read removal, and taxonomic classification. Bacterial composition and relative abundance were analyzed, with microbial abundance normalized as reads per million (rPM). Background contamination was controlled using CZ ID-derived models with Z-score-based filtering. Phage sequences were identified and quantified using the same mNGS pipeline by mapping reads against the NCBI viral database. Phage abundance was similarly expressed as rPM to track the longitudinal dynamics of the administered phages. Additionally, antimicrobial resistance genes were identified using the CZ ID AMR pipeline based on the CARD database v4.0.1 (https://card.mcmaster.ca).

### 2.7 Statistical analysis

GraphPad Prism 9.5.1 (GraphPad Software, CA, USA) was used for data visualization and statistical analysis. Experiments were performed with three biological replicates in technical triplicate, and data are presented as mean ± standard deviation (SD), with error bars indicating SD.

## 3 Results

### 3.1 Patient clinical history

An 87-year-old female with chronic obstructive pulmonary disease (COPD) presented with severe pneumonia caused by *Acinetobacter baumannii*, complicated by acute respiratory distress syndrome (ARDS) and renal failure. On admission, she exhibited progressive respiratory distress and impaired consciousness, requiring intensive care and mechanical ventilation. Initial laboratory evaluation showed markedly elevated inflammatory markers, including C-reactive protein (CRP) level of 172.56 mg/L and WBC count of 18.54 ×10^9^/L. Sputum cultures obtained on admission yielded extensively drug-resistant (XDR) *A. baumannii*, and broad-spectrum antibiotic therapy was initiated.

Despite prolonged and repeated antibiotic treatment after admission, the patient’s sputum cultures remained persistently positive for XDR *A. baumannii*, accompanied by progressive hypercapnia and worsening ventilatory impairment. This persistent infection indicated treatment failure, which highlighted the urgent need for an alternative therapeutic strategy to control the refractory infection and restore respiratory function.

### 3.2 Characterization of the *Acinetobacter baumannii* phage

**Figure 1:**
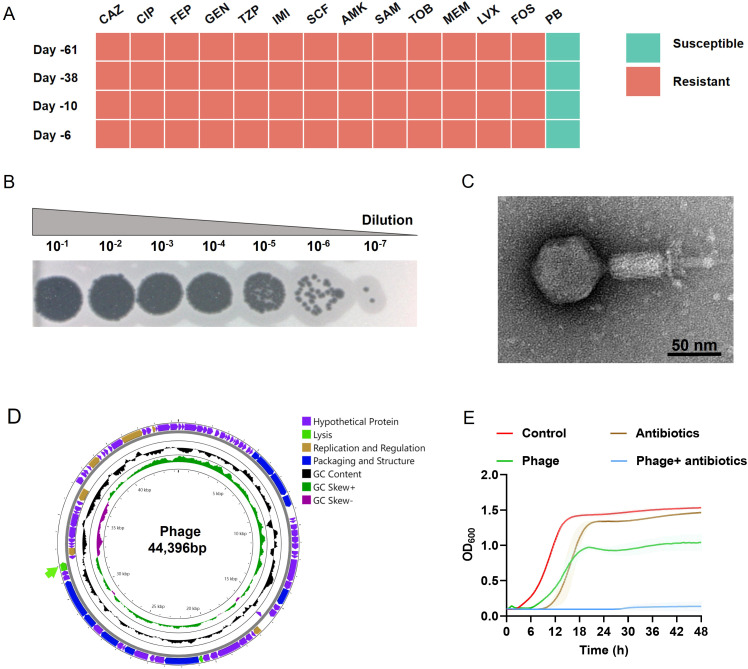
Characterization of the lytic *A. baumannii* phage. **(A)** Bacterial susceptibility to antibiotics (green, Susceptible; red, Resistant). CAZ, ceftazidime; CIP, ciprofloxacin; FEP, cefepime; GEN, gentamicin; TZP, piperacillin-tazobactam; IMI, imipenem; SCF, cefoperazone-sulbactam; AMK, amikacin; SAM, ampicillin-sulbactam; TOB, tobramycin; MEM, meropenem; LVX, levofloxacin; FOS, fosfomycin; PB, polymyxin B. **(B)** Visualization of phage plaque forming units (PFU) against the pre-therapy XDR *A. baumannii* isolated from patient sputum. **(C)** Transmission electron micrograph images of the isolated phage. **(D)** Genome map of phage. Endolysin genes were indicated by green arrow, DNA polymerase and RNA helicase were shown in yellow, and tail and head structural proteins were shown in blue. GC: guanine-cytosin. **(E)** Evaluating the antimicrobial effect of phage + antibiotics on XDR *A. baumannii* using time-kill assay. Phage was applied at 10^4^ PFU/mL (MOI = 0.1). Antibiotics were used at 0.25 × MIC, including fosfomycin (128 mg/L), amikacin (128 mg/L), and polymyxin B (0.5 mg/L). Data were presented as mean ± SD.

Antimicrobial susceptibility testing showed that the patient-derived XDR *A. baumannii* isolate remained susceptible only to polymyxin B (**Figure 1A**). However, prior intravenous polymyxin B therapy failed, likely due to its narrow therapeutic window and poor lung penetration (Imberti et al., 2010). Given that antibiotic therapy failed to resolve this refractory XDR *A. baumannii* infection, phage-antibiotic (fosfomycin, amikacin and polymyxin B) combinational therapy was employed for treatment. Above all, an XDR *A. baumannii* strain isolated from the patient’s sputum was used as the host strain for enrichment-based isolation of phage from hospital wastewater, followed by screening using double-layer agar plaque assays. Ultimately, phages producing clear plaques were purified through multiple rounds of plaque isolation, and a highly lytic phage was selected as the candidate for therapeutic application (**Figure 1B**). Transmission electron microscopy images revealed that this phage had an icosahedral head structure of 77.1 nm and a contractile tail of 120.6 nm × 27.8 nm (**Figure 1C**). Moreover, sequence similarity and wgANI analyses demonstrated that the phage was closely related to previously reported *Acinetobacter* phage WCHABP1 (NC_041966.1) but remained below the ICTV species demarcation threshold, indicating that the isolated phage was a new species within the class *Caudoviricetes*. Genomic sequencing results demonstrated that the phage genome comprised of 44, 396 base pairs, which revealed a series of genes encoding common phage-associated proteins, including DNA polymerase, RNA helicase (yellow), as well as tail and head structural proteins (blue). The endolysin gene encoding host lysis protein (green) was identified, and the absence of lysogenic, antimicrobial resistance and virulence genes was confirmed (**Figure 1D**). Together, genomic sequencing indicated that the phage possessed a complete genetic for bacterial infection, lytic replication, and gene expression.

Subsequently, phage amplification, production, and purification were performed to obtain therapeutic doses containing 5 × 10^9^ plaque-forming units (PFU)/mL. Quality control testing confirmed the sterility of the preparations and low endotoxin levels (3.38 endotoxin units [EU]/10^7^ PFU). The antibacterial activity against patient-derived XDR *A. baumannii* isolate was evaluated using time-kill assays. Phage monotherapy suppressed bacterial growth for 18 h, indicating rapid emergence of phage tolerance. Notably, phage + antibiotic therapy achieved sustained inhibition of bacterial growth for over 48 h and yielded an S_HSA_ score of 0.58, demonstrating a potent synergistic antibacterial effect between the phage and antibiotics (**Figure 1E**). These findings indicated that the wastewater-derived phage possessed strong lytic activity against XDR *A. baumannii*, and in combination with antibiotic regimen maintained durable bacterial suppression. Phage-antibiotic therapy exhibited great potential for the treatment of refractory XDR *A. baumannii* infections.

### 3.3 Clinical course following phage + antibiotic therapy

**Figure 2:**
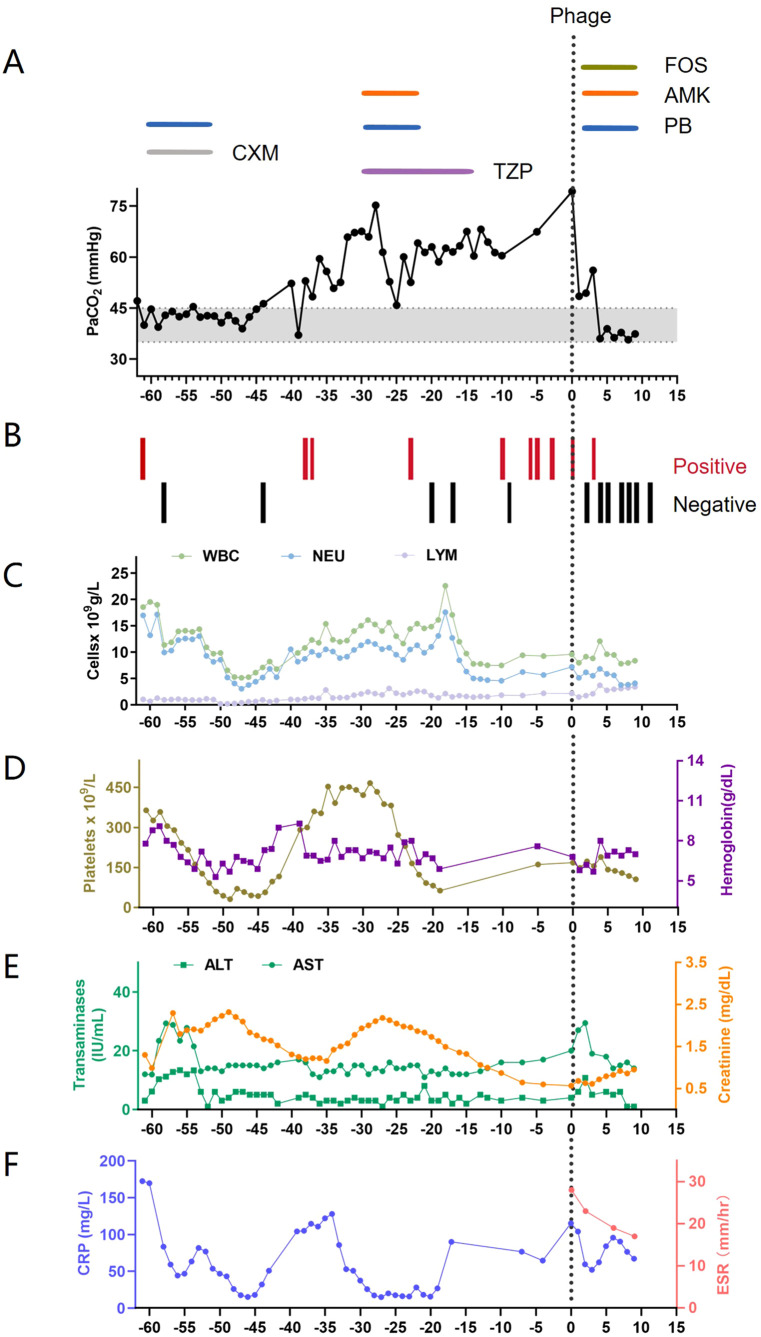
Clinical physiological summary from time of initial infection through phage + antibiotic therapy. **(A)** Timeline of antibiotic therapy and assessment of alveolar ventilation from the initial infection. Phage + antibiotic therapy was initiated on day 0. CXM, cefuroxime. TZP, piperacillin-tazobactam. PB, polymyxin B. AMK, amikacin. FOS, fosfomycin. PaCO_2_, arterial partial pressure of carbon dioxide. **(B)** Sputum culture results indicating recurrent *A. baumannii* infection using sole antibiotic therapy. Reduction of *A. baumannii* was observed following phage + antibiotic treatment. Red bars indicated positive culture result, and black bars denoted negative culture result. **(C, D)** Blood routine examination results. WBC, white blood cell, NEU, neutrophils, LYM, lymphocytes. **(E)** The levels of liver and kidney function indicators in peripheral blood. ALT, alanine transaminase. AST, aspartate transaminase. **(F)** The concentration of inflammatory indicators in peripheral blood. CRP, C-reactive protein. ESR, erythrocyte sedimentation rate.

Phage + antibiotic therapy was initiated on day 0. The patient received combined therapy consisting of nebulized phage (10^9^ PFU/mL, twice daily) and intravenous antibiotic regimen, including fosfomycin (8 g, every 8 hours), amikacin (0.2 g, every 12 hours), and polymyxin B (500, 000 U, every 12 hours) (**Figure 2A**). On the first day of treatment, the patient’s arterial partial pressure of carbon dioxide (PaCO_2_) decreased from a baseline of 79.3 mmHg to 48.5 mmHg and decreased to the normal range after day 4 (**Figure 2A**), indicating a rapid improvement in alveolar ventilation and resolution of hypercapnic respiratory failure. Sputum cultures for XDR *A. baumannii* were negative on day 2, rebounded on day 3, and remained consistently negative after day 4. This transient fluctuation suggested a possible sampling bias due to low bacterial load below the detection limit on day 2, probably driven by phage-mediated bacterial reduction. Ultimately, the sustained clearance after day 4 underscores the robust synergy between phage and antibiotic therapy (**Figure 2B**).

The safety profile of phage + antibiotic therapy were systematically evaluated. Importantly, the treatment was well tolerated, with no adverse events recorded throughout the study period. During phage + antibiotics therapy (day 0 to day 9), WBC count fluctuated within the range of 7.82-12.09 × 10^9^/L (normal range 4-10 × 10^9^/L), neutrophils within 3.77-7.23 × 10^9^/L (normal range 2-7 × 10^9^/L), and lymphocytes within 1.50-3.44 × 10^9^/L (normal range 0.8-4 ×10^9^/L) (**Figure 2C**). Although transient elevations above the upper limits were noted, no sustained leukocytosis or progressive inflammatory trend occurred, suggesting hematological stability and no therapy-induced inflammation. Additionally, platelet counts remained stable within the normal range (100-300 × 10^9^/L) throughout the combination therapy, in contrast to the severe thrombocytopenia observed during preceding antibiotic monotherapy. Hemoglobin (Hb) concentration from 6.8 g/dL on day 0 to 7.0 g/dL on day 9 (normal range 12–16 g/dL), with no evidence of treatment-related hematologic toxicity during the combination therapy (**Figure 2D**). Liver function tests remained stable during treatment (day 0 to day 9), with alanine transaminase (ALT) and aspartate transaminase (AST) values within the normal range (7–40 U/L and 0–45 U/L, respectively). Importantly, serum creatinine increased from a baseline of 0.56 mg/dL to a normal range of 0.95 mg/dL on day 9 (normal range 0.7-1.3 mg/dL), suggesting recovery from previously reduced creatinine levels, which may reflect improved renal function reservation and nutritional status after phage + antibiotic therapy (**Figure 2E**). C-reactive protein (CRP) decreased from 115.36 mg/L at day 0 to 67.33 mg/L at day 9 (normal range < 10 mg/L), and erythrocyte sedimentation rate (ESR) decreased from 28 mm/h to 17 mm/h (normal range < 20 mm/h), suggesting a reduction in systemic inflammatory burden associated with infection after phage + antibiotic therapy (**Figure 2F**). Altogether, phage + antibiotic therapy effectively resolved pulmonary XDR *A. baumannii* infection, improved clinical outcomes in ARDS and severe pneumonia, and demonstrated no renal or hepatic toxicity.

### 3.4 Bacteria reduction and antimicrobial resistance gene down-regulation

**Figure 3:**
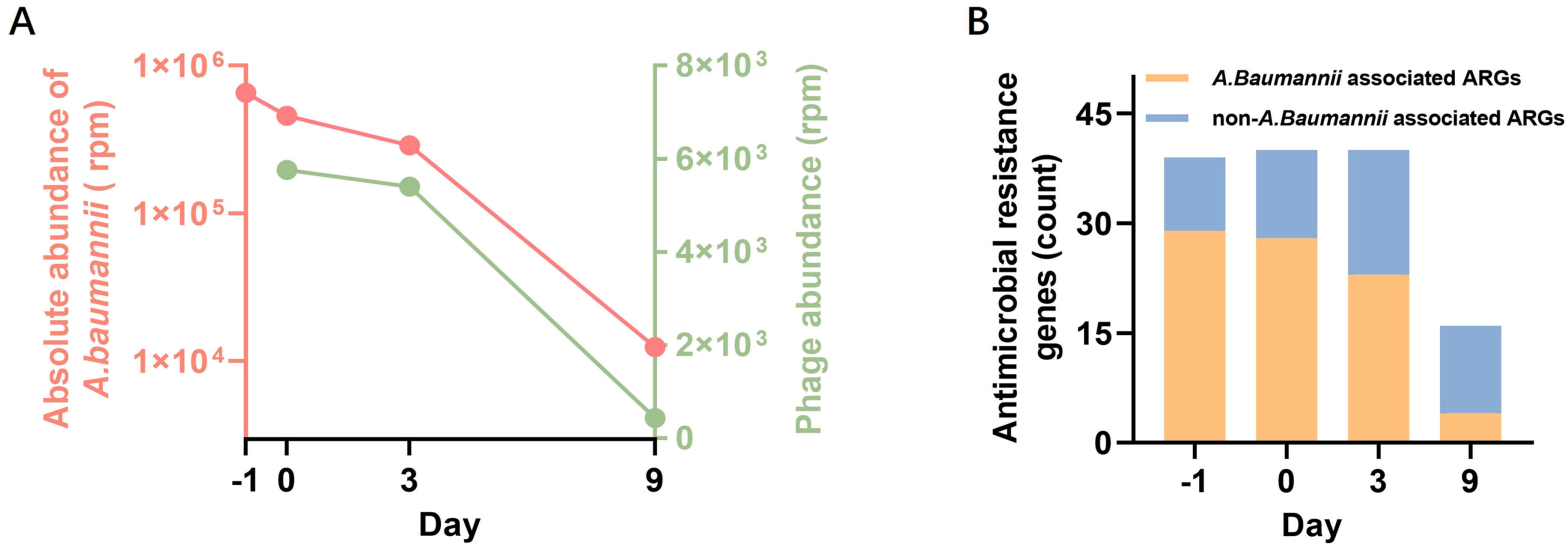
Dynamics of *A. baumannii* abundance and ARGs before and after phage + antibiotics therapy. **(A)** Absolute abundance of *A.baumannii* and phage in sputum samples. **(B)** Counts of *A. baumannii-*associated and non-*A. baumannii-*associated ARGs in sputum samples. ARGs, antimicrobial resistance genes.

Following phage + antibiotic therapy, we assessed the longitudinal changes of *A. baumannii* and phage absolute abundance in patient sputum, as well as ARGs count. Following nebulized phage administration, a high phage abundance was initially detected, with a subsequent reduction in XDR *A. baumannii* abundance, followed by a progressive decrease in phage abundance after treatment initiation (**Figure 3A**). As the *A. baumannii* burden decreased, phage abundance declined accordingly, consistent with host-dependent phage dynamics. Simultaneously, the counts of *A. baumannii*-associated ARGs progressively decreased from 29 before treatment to 4 on day 9 (**Figure 3B**). Together, phage + antibiotics therapy achieved rapid and effective XDR *A. baumannii* reduction accompanied by substantial reduction of ARGs burden in the lung.

## 4 Discussion

XDR *A. baumannii* infection has become a major therapeutic challenge in intensive care unit-associated infections, particularly in severe pulmonary infections (Deng et al., 2020). Switching to alternative antibiotics often results in transient suppression of bacterial growth (Andersson and Hughes, 2014), as resistant populations rapidly acquire adaptive mutations, leading to sustained treatment failure (Chung et al., 2022). Similarly, phage monotherapy is limited by the rapid emergence of phage-resistant variants, which enables bacterial escape from phage recognition and lysis, ultimately compromising therapeutic efficacy (Liu et al., 2020; Sanchez, 2011). Collectively, these limitations highlight fundamental shortcomings of monotherapies, underscoring the urgent need to explore phage-antibiotic combinations and to systematically evaluate their potential synergistic or antagonistic interactions. In this study, time-kill assays and synergy analysis demonstrated that phage-antibiotic therapy achieved synergistic bactericidal effects in 9 days through complementary mechanisms. The therapy enabled effective reduction of both XDR *A. baumannii* abundance and its associated resistance gene load in patient’s lung, thereby improving bacterial clearance and reduced antibiotic exposure. Phage-antibiotic synergy likely arises from antibiotic-induced physiological stress, such as impaired cell-wall integrity and altered membrane permeability, which may facilitate phage adsorption and infection (Bulssico et al., 2025; Gu Liu et al., 2020). In addition, bacterial evolutionary trade-offs associated with phage resistance could restore antibiotic susceptibility (Gu Liu et al., 2020). Phage-antibiotic combinational therapy provided a broadly applicable and practical strategy for the management of multidrug-resistant bacterial infections.

The enrichment and dissemination of ARGs in XDR *A. baumannii* is the primary cause of antibiotic therapy failure and drug resistance spread (Povolo and Ackermann, 2019). By promoting biofilm formation, enhancing virulence factor production and host injury (DiGiandomenico et al., 2012), the accumulation of ARGs increases bacterial stress tolerance, which drives persistent and refractory infections (Saenz et al., 2019; Lurie-Weinberger et al., 2025). Therefore, clearing antimicrobial resistance genes is the central challenge in treating drug-resistant bacterial infections. In our study, phage-antibiotic synergy not only efficiently suppressed viable drug-resistant bacteria, but also reduced *A. baumannii*-associated ARGs. Consistent with previous reports (Tchatchiashvili et al., 2026), our findings further supported the potential of phage-antibiotic therapy to enhance bacterial clearance, disrupt biofilms, and limited resistance progression. This effect may disrupt the molecular basis underlying the pathogenicity of drug-resistant bacteria, offering a novel clinical strategy for treatment failure, recurrence and prevalence of drug-resistant bacteria. In future, a prospective cohort study is required to systematically evaluate the efficacy, safety and generalizability of phage + antibiotic therapy to drug-resistant bacterial infections. And elucidating the dynamic evolution mechanisms between ARGs accumulation, pulmonary microbiome and phage-antibiotic dual resistance, will guide precision phage-antibiotic therapy and advance personalized treatments.

## Data Availability

The complete genome sequence of the therapeutic phage has been deposited in the Genome Sequence Archive (GSA, China National Center for Bioinformation/Beijing Institute of Genomics, Chinese Academy of Sciences) under accession number CRA041289. The data are publicly available at https://ngdc.cncb.ac.cn/gsa/.
